# The age of computational cardiology and future of long-term ablation target prediction for ventricular tachycardia

**DOI:** 10.3389/fcvm.2023.1233991

**Published:** 2023-09-14

**Authors:** Arsalan Moinuddin, Syed Yusuf Ali, Ashish Goel, Yashendra Sethi, Neil Patel, Nirja Kaka, Prakasini Satapathy, Ranjit Sah, Joshuan J. Barboza, Mohammed K. Suhail

**Affiliations:** ^1^ School of Sport and Exercise, University of Gloucestershire, Gloucester, United Kingdom; ^2^ Department of Biomedical Engineering, Johns Hopkins University, Balimore, MD, United States; ^3^ Department of Medicine, Government Doon Medical College, Dehradun, India; ^4^ PearResearch, Dehradun, India; ^5^ Department of Medicine, GMERS Medical College, Himmatnagar, India; ^6^ Global Center for Evidence Synthesis, Chandigarh, India; ^7^ Center for Global Health Research, Saveetha Medical College and Hospital, Saveetha Institute of Medical and Technical Sciences, Saveetha University, Chennai, India; ^8^ Department of Microbiology, Tribhuvan University Teaching Hospital, Institute of Medicine, Kathmandu, Nepal; ^9^ Department of Microbiology, Dr. D. Y. Patil Medical College, Hospital and Research Centre, Dr. D. Y. Patil Vidyapeeth, Pune, India; ^10^ Department of Public Health Dentistry, Dr. D.Y. Patil Dental College and Hospital, Dr. D.Y. Patil Vidyapeeth, Pune, India; ^11^ Escuela de Medicina, Universidad César Vallejo, Trujillo, Peru; ^12^ Moray House, University of Edinburgh, Edinburgh, United Kingdom

**Keywords:** ventricular tachycardia, catheter ablation, computational cardiology, metabolic sink theory, precision medicine

## Abstract

Ventricular arrhythmias, particularly ventricular tachycardia, are ubiquitously linked to 300,000 deaths annually. However, the current interventional procedure—the cardiac ablation—predict only short-term responses to treatment as the heart constantly remodels itself post-arrhythmia. To assist in the design of computational methods which focuses on long-term arrhythmia prediction, this review postulates three interdependent prospectives. The main objective is to propose computational methods for predicting long-term heart response to interventions in ventricular tachycardia Following a general discussion on the importance of devising simulations predicting long-term heart response to interventions, each of the following is discussed: (i) application of “metabolic sink theory” to elucidate the “re-entry” mechanism of ventricular tachycardia; (ii) application of “growth laws” to explain “mechanical load” translation in ventricular tachycardia; (iii) derivation of partial differential equations (PDE) to establish a pipeline to predict long-term clinical outcomes in ventricular tachycardia.

## Graphical Abstract

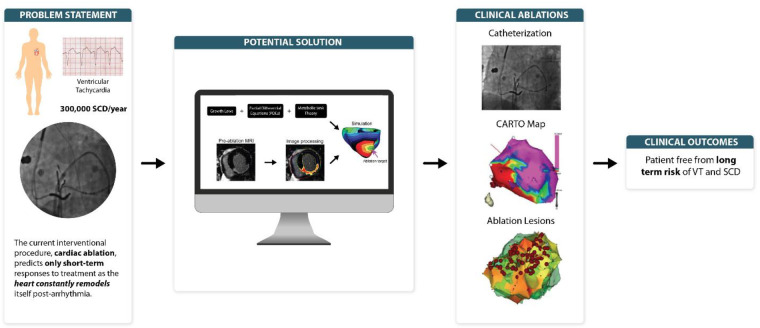
 Predicting long-term ablation targets for ventricular tachycardia (1–7).

## 1. Introduction

Ventricular arrhythmias, a subset of cardiovascular disease (CVD), is ubiquitously linked with 300,000 sudden cardiac deaths (SCD) each year, an incidence rate of 53 per 100,000 population (5.6% of all-cause mortality) (1). Its two variants: ventricular tachycardia and ventricular fibrillation, are associated with SCD that require an instant interventional procedure—the *cardiac ablation*—to amend arrhythmia, consequently reducing SCD and stroke outcome (2). Clinically, to perform an ablation procedure, cardiologists have to rely on computational methods, which typically integrate a plethora of medical data (cardiovascular parameters, radiographs, etc.). This provides a diagnostic framework to predict the outcome of interventional cardiac ablation procedures. These computational methods only predict short-term responses to treatment, as the heart constantly remodels itself with either disease progression or regression. To assist in the design of computational methods which focus on long-term simulation, this review postulates three prospectives that may plug in gaps in the literature (3–5). The main objective is to propose computational methods for predicting long-term heart response to interventions in ventricular tachycardia. Following a general discussion on the importance of devising simulations predicting long-term heart response to interventions avoiding multiple procedures on patients, each of the following is discussed: (1) Application of “metabolic sink theory” to elucidate the “re-entry” mechanism of ventricular tachycardia (VT). (2) Application of “growth laws” to explain “mechanical load” translation in ventricular tachycardia (VT). (3) Derivation of partial differential equations (PDE) to establish a pipeline to predict long-term clinical outcomes in ventricular tachycardia (VT)—the upsurge of Personalized Medicine, which uses individualized patient data to unfold concealed diagnosis and predict outcome of specific interventional procedures on that particular patient.

## 2. Importance of devising simulations—predicting long-term response to interventions

Ventricular arrhythmia-induced SCD remains a leading cause of death in the west. Whilst the management approach encompasses anti-arrhythmia drugs, ablation therapy, and implantable cardioverter-defibrillators (ICDs), ICDs still remain the most effective method to reduce SCD incidence (ICD vs. non-ICD hazard ratio 0.69, *p* = 0.016) (6). Six out of eight randomized trials evaluating the prophylactic use of ICD via Markov model found cost-effectiveness ratio below $100,000 per QALY comparable to other interventions. However, as stated by Huikuri et al., large-scale deployment of prophylactic ICD is still cost-ineffective (up-front cost of ∼$20,000 for each device) compared to its counterpart, the coronary artery bypass graft (CABG) procedure (5). The current SCD predictors: (1) Echocardiography, and (2) Contrast Angiography lack specificity, and SCD incidents are primarily observed in patients without ICDs. Thus, there is a pressing need to understand the mechanistic basis of arrhythmogenesis in ventricular arrythmia patients, specifically who are at high risk of SCD (5).

Current ablation procedures use computer simulations that apply a general time-based approach pivoted around the left ventricular ejection fraction (LVEF) to predict arrythmia risk (4, 7). However, there exists ample ambiguity in relation to post-infarct scars, and the time taken for complete scar healing post ventricular arrhythmias. Further, as Trayanova lab elaborated short-term approach is suboptimal as patients often undergo failed ablations that require them to undergo multiple procedures, posing additional risk and discomfort to them (4, 7). This certainly leads to an imperative requirement for long-term prediction of ablation targets to eliminate need of repeating these procedures.

Currently, the science focus of most funding agencies is to back innovative research strategies which have scientific rigor and to look out for cost-effective disease interventions. This aligns well with our preposition design of personalized computational models, specifically for the long-lasting ventricular arrhythmia prognosis. These models not only resemble diseased hearts and can simulate mechanistic relationships of cardiac pathologies, but also personalize the interventions specific to the patient's heart condition as previously shown by Trayanova et al., Kroon et al., and Bifulco et al. (4, 8–10).

## 3. Application of “metabolic sink theory” to elucidate the “re-entry” mechanism of ventricular tachycardia

The postulated mechanisms in the pathogenesis of VT include triggered activity, altered automaticity, and re-entry (11). In this section, we will focus on VT's “re-entry” mechanism which presents a novel “metabolic sink theory” to explain the arrhythmia mechanism in the heart (12). Briefly, O'Rourke and colleagues' working hypothesis was that metabolic stress causes a reduction in ATP/ADP ratio in diseased cardiomyocytes, which in turn activates sarcolemmal K-ATP currents, resulting in the elimination of action potential in the damaged cardiomyocytes (13). However, the healthy intact cells act as cellular regions of in excitability termed as “metabolic sinks” of current, which initiate heterogeneous conduction and, consequently, initiate “re-entry” arrhythmias (13, 14).

**Figure 1:**
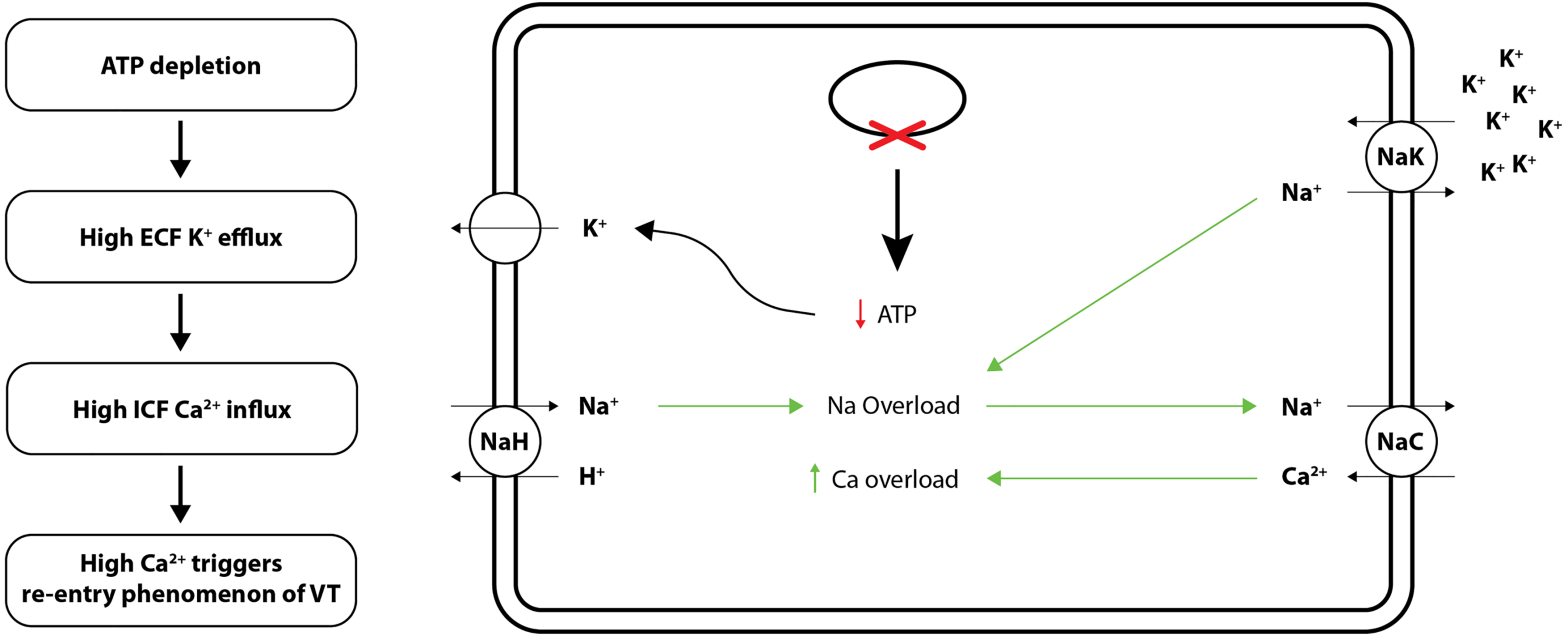
Cellular mechanisms (ion channels and electrolyte exchange) underlying “metabolic sink theory” of re-entry phenomenon of VT. ATP, adenosine triphosphate.

To elaborate, “metabolic sink theory” suggests that long-term pressure and volume changes in VT patients, harbour high Ca^+^ ions in cardiomyocytes, forming “metabolic” sinks of current. As mentioned above, O'Rourke's lab created the first cardiac cell model to link: (1) ion fluxes, (2) energy-consuming reactions (e.g., myosin ATPases), and (3) energy production. With respect to hemodynamic homeostasis, they have reported that growth and remodelling, i.e., thickening, and lengthening of cardiac myocytes translates into electrophysiological alterations that could lead to the “re-entry” phenomenon in VT (13, 14). Further, ischemia in VT translates into metabolic stress in the form of free radicals, which shortens cardiomyocyte action potentials. As per the “energy starvation theory”; because ATP is required for normal systolic and diastolic contractile function, ATP concentration and consequently the ATP/ADP ratio falls in failing human heart conditions such as the ventricular tachycardia (VT) as ATP generating pathways, e.g., the creatine kinase (CK)-phosphocreatine (PCr) system fails out. A decrease in the cellular ATP/ADP ratio activates K+ATP channels, resulting in significant ECF K+ outflow. Of note, low K^+^ concentration inside the cells also stimulates influx of Ca^+^ ions from intracellular fluid (ICF) (13). The primary outcome of “metabolic sink theory”; model is depicted in Figure 1.

The proposition is innovative because it represents a new and substantive departure from the status quo. It utilizes the “metabolic sink theory” to define the re-entry mechanism of ventricular arrhythmogenesis via the development and innovation of cardiac simulation equations. It is driven by the fact that re-entry mechanisms underlying arrhythmogenesis are not comprehensible by direct experimental observation. The reason being the overlapping emission spectra of fluorescent indicators make it hard to record cardiac myocyte ATP/ADP ratio and membrane potential currents simultaneously. Additionally, it provides an understanding of the multifaceted relationship between metabolic and electrophysiological activity. Importantly, the insights gained might improve the selection criteria for ICD candidates and lead to new pharmacological therapies to combat arrhythmia.

## 4. Application of “growth laws” to explain “mechanical load” translation in ventricular tachycardia (VT)

In response to post-VT cardiac injury and fibrosis, cardiomyocytes change their electrical activity in order to adapt to the heart's changing metabolic needs. Recent clinical studies have shown that a severe congestion and a greater myocardial stretch in heart failure (HF) patients with ICDs, measured using the HeartLogic (Boston Scientific) Index and indicated by an increased third heart sound amplitude, facilitates ventricular ectopy that can trigger VT (15). Thus, altered mechanical signals (stress and strain) convert into unique hemodynamic pressure-volume changes that efficiently pump blood throughout the body. We suggest that computer-generated simulation models can convert this mechano-adaptation to self-generated hemodynamic by using “growth laws”—a series of equations that empirically produce pressure and volume load *in vivo* by enforcing artificial stretches and strains (16). Several data reported these phenomenological growth laws to model growth response following VT, albeit the underlying mechanisms underpinning cardiac hypertrophy post VT remain elusive. For instance, Rodriguez et al. postulated a set of equations, they termed as growth laws, which specify cardiac dimensions, residual stresses, and the repeated cyclic loading response post VT injury. Further, Grossman et al. estimated that peak systolic stress acts as a stimulus for concentric cardiac wall hypertrophy, whilst end-diastolic fiber strain stimulate eccentric hypertrophy. Furthermore, using independent mechanical inputs and feedbacks linked to the tailored *in vivo* load will aid in the development of simulation equations that will anticipate information regarding cardiac musculature remodelling (17).

Functionally, heart muscles undergo pressure and volume adaptations in terms of growth and remodelling to counter VT. These adjustments allow heart's residual healthy tissue to enable pump blood throughout the body to meet the organ systems’ oxygen perfusion demand. Specifically, heart's blood pressure and volume control system change its geometry to adapt to this new mechanical load by transferring mechanical stress and strain signals to the heart walls. Of note, when ventricles are subjected to increased pressure and/or volume overloading, “growth laws” predict how much would be the compensatory concentric growth (thickening of individual myocytes) and/or eccentric growth (lengthening of myocytes), respectively. A typical example was Taber's embryonic growth model of heart's alterations in ventricular chambers during foetal growth. Their group acknowledged that pre-natal hyperplasia (increase in cell size) and post-natal hypertrophy (increase in cell number) in response to volume and pressure load respectively was similar to structural changes in human heart (18). They argued that general principles of biomechanical growth laws applicable to skeletal muscles and muscular arteries hold true for cardiac muscle growth. Kroon et al. (2009) further implicated simulation of soft-tissue growth laws in their ventricular growth model (9) which operate on the tenet of elastic fiber strain at the end-diastole phase of the cardiac cycle. Lastly, Göktepe et al. (2010) adopted a similar approach to the sarcomerogenesis model of eccentric and concentric cardiac growth which was centred around the end-systolic and -diastolic simulation models (19). Collectively, growth laws appear to have enormous potential for recognizing the endogenous cardiac regeneration archetype and repair by providing clinically relevant predictive models of cardiac growth.

## 5. Derivation of partial differential equations (PDEs) to establish a pipeline to predict long-term clinical outcomes in VT—the upsurge of personalized medicine and computational cardiology

As interventional cardiologists face the equivocal challenge of performing the specific interventional procedure for a particular patient, precision medicine paved their way out (4). Personalized medicine uses the current understanding of human cardiac physiology and biophysics to integrate a variety of medical data from individualized patients (radiographs, cardiovascular parameters, etc.). It reveal: (1) concealed diagnostics, (2) provide a common diagnostic framework that would otherwise be obscured, and (3) predict the outcomes of the various interventional cardiac procedures (4, 20). Computational cardiology provides computational tools in cardiology research that analyze mathematical models of diseased cardiac physiology and use patient-specific clinical data to collectively propose most appropriate intervention for the patient (4).

To construct a 3-dimensional (3D) computer model of an individual patients diseased heart, first LGE-MRI scans are acquired to reconstruct the heart's geometry and fiber/sheet orientation. LGE-MRI scans, in particular—denote a specific variant of cardiac resonance scans that are meant to estimate myocardial regional scar formation and/or fibrosis (17). Specifically, late gadolinium enhancement (LGE) is built around the shortening of the T1 weighted image (pulse sequence in MRI) and gadolinium-based contrast agents, giving a brighter signal and best contrast to the T1 weighted image (17). The next step is the contouring of ventricular walls utilizing the landmark points; followed by the construction of the 3D ventricular structure. Identification of the infarct zones is the first key stage in developing a pipeline to model a patient's heart. Infarct zones often referred to as grey zones (GZ), are detected through segmentation into the existing structure (21). Assignment of human cellular and tissue electrophysiological properties to both the normal and the fibrotic tissue (scar, GZ) is the next step in the model construction line-up (4). The scar zones are treated as non-conductive zones of no electrical activity. The electrical conductance in all the zones mentioned above is simulated by applying the reaction-diffusion equation (describes current flow through cardiac cells that are electrically connected, allowing for a continuum representation) This finite element method applicable in a variety of biological situations and offering solutions to these equations demonstrate a graphic pattern and traveling waves in these biological specimens. Collectively, three factors contribute to designing 3-dimensional (3D) computer model of an individual patients diseased heart: (1) patient's personalized ventricular geometry, (2) resultant remodelling of ventricular chamber in response to the existing disease, and (3) electrical properties of cardiomyocytes.

Classical ventricular tissue action potential models like Courtemanche et al. (22) and ten Tusscher et al. (23) describe in detail how a biophysical process is modelled into PDEs. These PDE models have been developed into powerful software engineering platforms like openCARP for use in the clinic (24). These PDE models have been validated with *in vitro* cell experiments, animal experiments and human studies (25–27).

**Figure 2:**
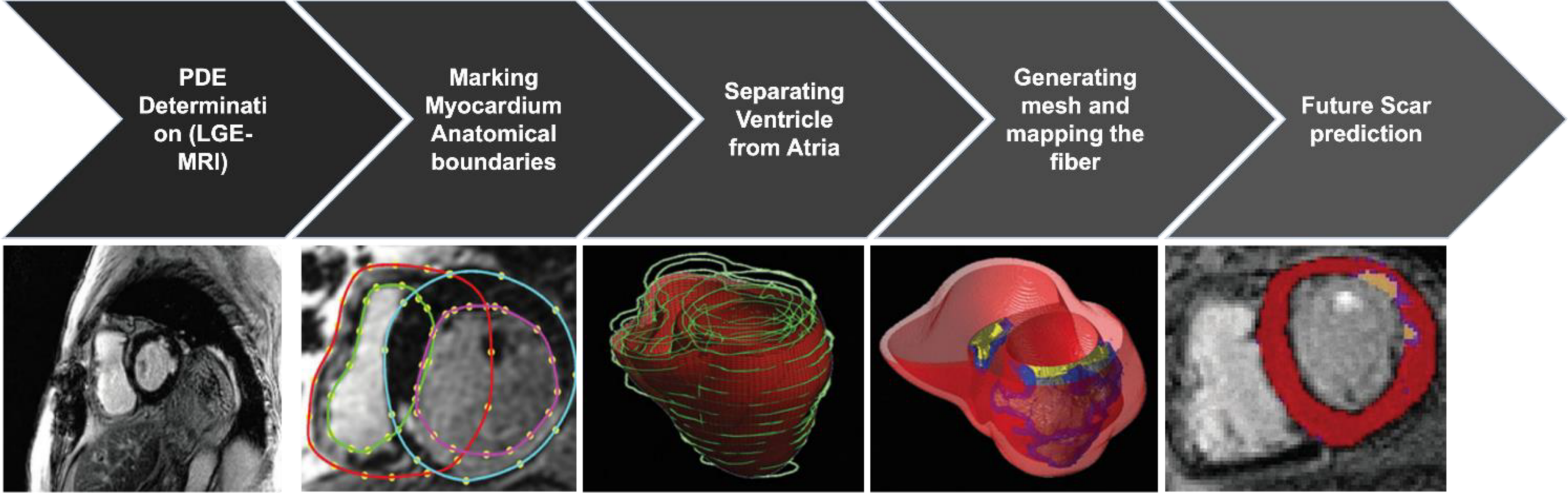
The pipeline for image-based reconstruction of heart geometry and structure to generate patient specific ablation strategy to prevent long term VT and SCD risk (3, 7).

A schematic of the partial differential equations (PDE) “pipeline” for image-based reconstruction of heart geometry and structure is outlined in Figure 2: (1) Extracting the myocardium boundary from the suspension media by detecting the image edges and then using a region growing algorithm. (2) Separating the ventricular tissue from the atria using a closed spline curve fitting method. (3) Generating the computational mesh using an efficient octree-based spatial discretization of myocardial volume. (4) Mapping the fiber and sheet orientations. (5) Combining growth laws and classical cardiac action potential PDE models to simulate and predict the long-term VT episodes and SCD risk. (6) Generating final ablation strategy based on simulations to present to the clinical team to perform ablations on the patient heart during the procedure (28).

With the rapid advancement in catheter design and technology [for instance, microelectrode electroanatomic mapping (EAM) for higher resolution maps] to get accurate information about the patient heart's electrophysiological condition invasively as well as the development of powerful algorithms like coherent mapping, LAT hybrid module and map replay modules from CARTO®3 (Biosense Webster Inc., Diamond Bar, CA, USA) that provide information critical to accurate VT ablation, these powerful tools can be used to validate our proposed PDE models (29, 30). In the future, with more powerful computational resources and development in the field of AI-accelerated modelling, our proposed PDE models can be deployed to work in real-time, while simultaneously incorporating information from the EAM mapping systems and presenting the clinician with the best VT ablation strategy for each patient for long-term freedom from arrhythmia (31).

### 5.1. Manuscript's scope

Whilst some basic context is provided, this review is not intended to provide coding intricacies or comprehensively discuss mechanistic pathways at the cellular level. For clarity, the intention of this review is to provide three key considerations to assist in devising simulations of long-term heart response to interventions avoiding multiple ablation procedures on patients.

## 6. Conclusion

While current interventional procedure—the *cardiac ablation*—envisage only short-term responses to VT, clinicians have begun to focus on computational methods predicting long-term post arrhythmia responses. To assist in this design, this review postulated the existing gaps in literature. First, the “metabolic sink theory” define the re-entry mechanism of ventricular arrhythmogenesis via development and innovation of cardiac simulation equations. Second, computer-generated simulation models can be haemodynamically translated by applying the “growth laws”—a system of equations that experimentally induce pressure and volume load *in vivo* by enforcing artificial stretches and strains. Lastly, the derivation of partial differential equations (PDE) “pipeline” for image-based reconstruction of heart geometry and structure will pave way for personalized medicine. The current ventricular tachycardia predictors, particularly in patients who are at high risk of SCD lack specificity. Our proposed mechanistic basis of arrhythmogenesis not only predict long-lasting ventricular tachycardia prognosis, but also has the potential to offer insight into personalized interventions specific to individual patient. Although, we are far-flung from predicting clinical outcomes in ventricular tachycardia (VT), these perspectives can still accelerate the long-term VT prediction quest.

## Data Availability

The original contributions presented in the study are included in the article/Supplementary Material, further inquiries can be directed to the corresponding authors.
